# Canine ACL rupture: a spontaneous large animal model of human ACL rupture

**DOI:** 10.1186/s12891-021-04986-z

**Published:** 2022-02-05

**Authors:** Emily E. Binversie, Brian E. Walczak, Stephanie G. Cone, Lauren A. Baker, Tamara A. Scerpella, Peter Muir

**Affiliations:** 1 Comparative Orthopaedic & Genetics Research Laboratory, School of Veterinary Medicine, University of Wisconsin-Madison, Madison, WI 53706 USA; 2 Department of Orthopedics & Rehabilitation, School of Medicine & Public Health, University of Wisconsin-Madison, WI 53726 Madison, USA; 3 Department of Mechanical Engineering, University of Wisconsin-Madison, Madison, WI 53706 USA

**Keywords:** ACL rupture, Human, Dog, Spontaneous animal model, Shared features, One Health

## Abstract

**Background:**

Anterior cruciate ligament (ACL) rupture in humans is a common condition associated with knee pain, joint instability, and secondary osteoarthritis (OA). Surgical treatment with an intraarticular graft provides reasonable outcomes at mid and long-term follow-up. Non-modifiable and modifiable factors influence risk of ACL rupture. The etiology, mechanobiology, causal biomechanics, and causal molecular pathways are not fully understood. The dog model has shared features of ACL rupture that make it a valuable spontaneous preclinical animal model. In this article, we review shared and contrasting features of ACL rupture in the two species and present information supporting spontaneous canine ACL rupture as a potentially useful preclinical model of human ACL rupture with a very large subject population.

**Results:**

ACL rupture is more common in dogs than in humans and is diagnosed and treated using similar approaches to that of human patients. Development of OA occurs in both species, but progression is more rapid in the dog, and is often present at diagnosis. Use of client-owned dogs for ACL research could reveal impactful molecular pathways, underlying causal genetic variants, biomechanical effects of specific treatments, and opportunities to discover new treatment and prevention targets. Knowledge of the genetic contribution to ACL rupture is more advanced in dogs than in humans. In dogs, ACL rupture has a polygenetic architecture with moderate heritability. Heritability of human ACL rupture has not been estimated.

**Conclusion:**

This article highlights areas of One Health research that are particularly relevant to future studies using the spontaneous canine ACL rupture model that could fill gaps in current knowledge.

## Background

Anterior cruciate ligament (ACL) rupture is a common condition associated with knee pain and life-changing loss of function. The estimated incidence in humans is ~ 13.5–75/100,000 persons per year [1]. Primary ligament repair has historically proven unsuccessful and current surgical treatment involves ACL graft reconstruction, although there is recent growing interest in a bridge-enhanced primary repair technique [2, 3]. There is a ~ 50% risk of knee osteoarthritis (OA) after ACL rupture that is not influenced by surgical treatment [4]. It has been recognized for many years that familial predisposition to ACL rupture exists [5].

Animal models are important for investigating biological mechanisms and developing new treatments. Dogs, goats, sheep, pigs, and rabbits have all been used as large animal models for ACL research. Readers are referred to Bascuñán et al. for a comparison of the strengths and limitations of these large animal models used for human ACL rupture [6]. Client-owned companion animals that develop disease spontaneously are vital to One Health research. One Health is the intersection of human health, animal health and their shared environment which considers shared risk factors, biology, and disorders while also recognizing the impact of important differences. Shared environment and high prevalence of spontaneous cranial cruciate ligament rupture makes the client-owned dog a widely accessible preclinical animal model of human ACL research.

The morphology of the canine stifle (knee), including the cruciate ligament complex consisting of the cranial (anterior) and caudal (posterior) cruciate ligaments (PCL), and the meniscal cartilages [7] is proportionally like the human knee. An intercondylar notch (ICN) and posterior tibial slope (PTS) is present in both species. For consistency, human anatomic terminology will be used to describe both species in this article.

Initially considered a traumatic injury in dogs, it is now accepted that mid-substance ACL rupture is a consequence of progressive fiber damage [8]. In dogs, ACL rupture incidence is up to ~ 2610/100,000 dogs per year in high-risk breeds (e.g., Newfoundland, Rottweiler, Labrador Retriever, Bulldog and Boxer) [9, 10]. In 1973, experimental ACL transection was performed in laboratory dogs as a model of OA [11]. However, artificially induced ACL transection models may not completely replicate the biological environment and pathologic tissue changes associated with spontaneous disease.

Canine ACL rupture has a similar natural history to the human condition, as meniscal damage and OA typically develop [12–14] (Table 1). Incomplete or partial ACL rupture occurs in both species [15, 16]. Specific operative techniques for surgical treatment differ in humans and dogs [17, 18]. Clinical presentation, diagnostic imaging, and treatment for both species are summarized in Table 2. This review aims to highlight shared features of ACL rupture between the two species and underscore possible research opportunities where this model could be used to close gaps in knowledge, particularly regarding genetics, development of post-traumatic OA, and non-surgical treatment of ACL rupture and knee OA.

**Table 1:** Epidemiology, etiology, and pathology of canine and human anterior cruciate ligament (ACL) rupture

Parameter	Canine ACL rupture	Human ACL rupture
Heritability	0.27–0.48 in high-risk breeds	Unknown
ACL bundles	Anteromedial and posterolateral	Anteromedial and posterolateral
Sex	Increased risk with neutering	Increased risk in athletic females
Incidence	Up to ~ 2610/100,000 dogs per year in high-risk breeds	~ 13.5–75/100,000 persons per year
Pathophysiology	Mainly non-contact rupture	Mainly non-contact rupture
Prodromal fiber rupture	Typical	Unknown
Contralateral ACL rupture	Up to 73% of cases	Up to 12.5% of cases
Secondary meniscal damage	Typical	Typical
Development of knee OA	Associated with ACL fiber rupture, often precedes knee instability	Multifactorial, often follows ACL rupture
Epidemiological risk factors	Breed, neutering, obesity	Increased risk in women. Activity that increases shoe playing surface friction and torsional forces
Molecular pathways	Altered ECM homeostasis and synovitis	Altered ECM homeostasis

*ACL* anterior cruciate ligament, *ECM* extracellular matrix, *OA* osteoarthritis

**Table 2:** Clinical, radiographic and treatment parameters for canine and human anterior cruciate ligament (ACL) rupture

Parameter	Canine ACL rupture	Human ACL rupture
Diagnosis	Clinical and radiographic	Clinical and radiographic
Symptoms	Knee pain and instability	Knee pain and instability
Screening test	Anterior drawer, tibial compression	Anterior drawer, Lachman, pivot-shift
Radiographic effusion		
Before diagnosis	Yes	No
At diagnosis	Yes	Yes
After diagnosis	Yes	Yes
Radiographic OA		
Before diagnosis	Typical	Atypical
At diagnosis	Typical	Atypical
After diagnosis	Yes	Frequently at long-term follow-up
Detection of ACL fiber rupture and secondary signs	MR imaging	MR imaging
Prediction of disease progression from incomplete to complete ACL rupture	Knee radiography	None
Arthroscopic ligament findings	Fiber rupture in both ACL bundles and PCL	Fiber rupture in both ACL bundles
Other arthroscopic findings	Synovitis, articular cartilage fibrillation and softening, meniscal tear, periarticular osteophytes	Synovitis, articular cartilage fibrillation and softening, meniscal tear
Histological changes in the ACL	Loss of collagen fibers and fiber crimp, chondroid transformation of ligament fibroblasts	Loss of collagen fibers and fiber crimp, chondroid transformation of ligament fibroblasts
Conservative treatments	Physiotherapy, activity modification, knee brace occasionally	Physiotherapy, activity modification, knee brace
Surgical treatments	Stabilization by tibial osteotomy or extracapsular suture	Stabilization by ACL reconstruction with intraarticular graft. Repair of proximal ACL avulsion, extraarticular augmentation^a^

*ACL* anterior cruciate ligament, *PCL* posterior cruciate ligament, *OA* osteoarthritis, *MR* magnetic resonance; ^a^Less commonly used/investigational

### Comparative anatomy

**Fig. 1:**
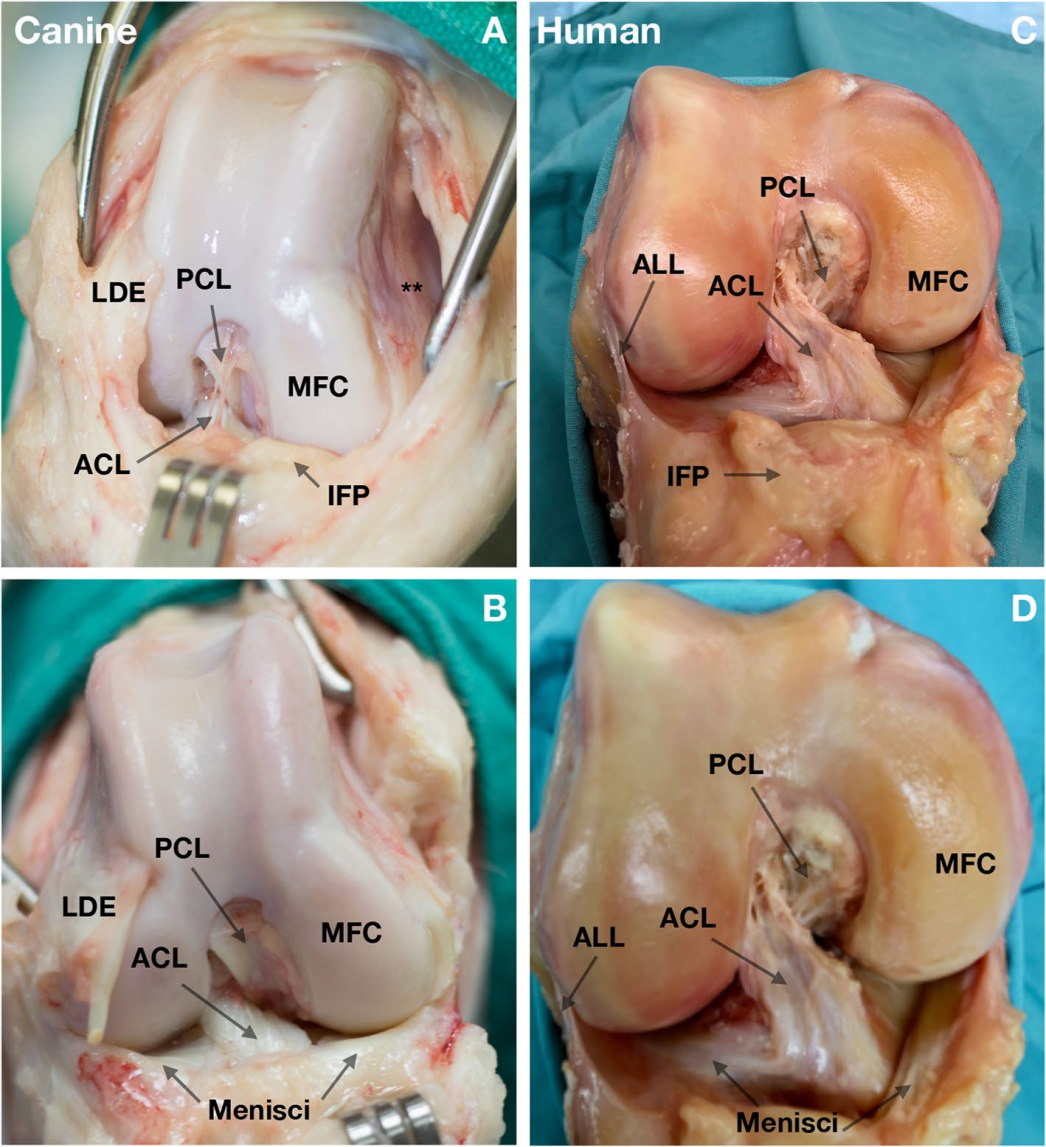
Anatomical features of the dog and human knee. **A**, **B **The right knee of a dog. **C**, **D** The right knee of a human. Anatomic features including an anterior cruciate ligament (ACL), posterior cruciate ligament (PCL), infrapatellar fat pad (IFP), lateral and medial femoral condyles (MFC) and lateral and medial menisci are similar. An important difference between the dog and human knee is the lack of an anterolateral ligament (ALL) in the dog. **A** Medial femoral pouch (**). In **B**, the view laterally was improved by transecting the long digital extensor (LDE) tendon

The canine and human knee has comparable intraarticular features, including the ACL, PCL, ICN, femoral condyles, menisci, and tibial plateau (Fig. 1). The infrapatellar fat pad and the synovium surrounding the cruciate ligaments provide a similar rich vascular envelope [19, 20]. The anterolateral ligament (ALL) is absent in the dog.

The primary biomechanical function of the ACL is to resist anterior tibial translation, internal tibial rotation, and hyperextension [19, 21]. The anteromedial bundle of the human ACL is taut during flexion [22] while in the dog the anteromedial bundle is taut during extension [23]. ACL tensile properties are similar in both species with comparable mean ultimate loads (2160 ± 157 N) in humans and (1867 ± 324 N) dogs [24, 25] despite a smaller body size. ACL stiffness is also similar in humans (242 ± 28 N/mm) and dogs (201 ± 41 N/mm) [24, 25].

Tibial plateau morphology in quadrupeds and bipeds is different. An osteological study reported mean PTS of 6.9 ± 3.7° along the medial condyle and 4.7 ± 3.6° along the lateral condyle in humans [26]. In dogs, PTS 24.0 ± 3.2° medially and 25.5 ± 3.8° laterally [27]. A more flexed standing angle at the knee is found in dogs and a higher PTS such that functional PTS is parallel to the ground [28]. A limitation to the canine ACL model is the dog’s flexed weight-bearing knee may not precisely model the same high compressive forces experienced in a weight-bearing extended human knee due to differences in gait biomechanics and anatomy.

#### Research opportunities

Progressive degradation of ACL biomechanical properties as incomplete ACL rupture develops could be an important area of future research.

### Etiology

In humans, approximately 70% of ruptures arise from non-contact injury typically after a sudden change in velocity and direction with a planted foot [29]. The proportion of non-contact ruptures in dogs is higher (~ 99% of cases). ACL rupture occurs when plastic tissue damage occurs from excessive loading. Rupture risk is influenced by ACL structure, composition and biomechanics, knee anatomy, and modifiable factors. It is now recognized that repeated submaximal knee loading with subfailure fiber rupture developing gradually over time explains some ruptures in humans [30] like dogs.

Incomplete ACL ruptures represent 10–27% of cases in humans [31], with similar OA progression to that of complete ACL rupture [32]. In the dog, incomplete ACL rupture typically progresses to complete rupture with an unstable knee [33].

Human patients with an ACL rupture are twice as likely than individuals without ACL rupture, to have a relative with ACL rupture [5], but heritability has not been determined. In dogs, ACL rupture is moderately heritable [34].

#### Research opportunities

Current knowledge suggests that development of subfailure fiber rupture and the associated inflammatory response within the knee joint is another important area for future comparative research. Knowledge of the genetics of canine ACL rupture is much advanced relative to human ACL rupture and translational genetic studies represent another substantial research opportunity.

### Non-modifiable factors

#### Age

In humans, ACL rupture risk is increased in adolescents and young adults [29]. In the Labrador Retriever, peak age of onset is 4 years with few dogs presenting over the age of 8 years [35]. Large breed dogs and dogs with bilateral ACL ruptures present at a younger age [9, 36, 37].

#### Sex and hormonal factors

In humans, young female athletes are at highest risk of ACL rupture, including contralateral ACL rupture [29]. Sex hormone effects on knee valgus and anterior-posterior laxity may influence risk of injury. Estrogen, progesterone, and androgen receptors are found on human ACL fibroblasts [38, 39]. Androgen receptors have also been identified in canine ACL fibroblasts [40]. Increasing estrogen and ACL laxity in the preovulatory phase may influence knee valgus and external rotation and associated ACL strains in women [41]. However, women often present with ACL ruptures during the early follicular and late luteal phase with low estrogen and progesterone [42]. ACL rupture risk in dogs is increased by ovariohysterectomy or castration [43]. Prevalence of ACL rupture is higher in neutered dogs, particularly when neutered at a young age [36]. In dogs, neutering may influence PTS [44].

#### ACL physiology and size

A smaller ACL volume is associated with increased risk of ACL rupture in men [45]. A larger ACL volume is correlated with higher ligament yield load and higher load to failure in dogs [46]. High-risk breeds have higher laxity, higher collagen turnover, lower enthalpy of denaturation and smaller collagen fibril diameters [47, 48], but ACL cross-sectional area is like low-risk breeds [47].

#### Intercondylar notch

Within the femoral ICN, the ACL and PCL twist around each other. A stenotic ICN may cause ACL impingement and promote fiber rupture [49], with associated increased risk of ACL rupture in both species [49, 50]. In women, decreased ICN width is a risk factor for ACL rupture and graft rupture [45]. In high-risk dog breeds, ACL impingement by the ICN is associated with increased collagen remodeling and reduced ACL structural integrity [49]. However, in a study of Labrador Retrievers, ICN stenosis did not contribute to risk of canine ACL rupture in vivo [51].

#### Proximal tibial morphology

Studies in humans and dogs have shown that a higher PTS increases ACL load when larger axial compression forces are applied [52, 53]. In humans, increased PTS influences risk of both ACL rupture and ACL graft rupture [54]. In dogs, higher PTS also contributes to risk of ACL rupture [51] and matrix degeneration in the ACL over time [55]. A small relative tibial tuberosity width in dogs also increases ACL rupture risk [56].

#### Axial rotation at the hip and knee

In humans, limited internal rotation at the hip increases peak ACL strain during pivot landings [30]. Dynamic alignment characterized by hip adduction, internal rotation, and increased knee abduction increases risk of ACL rupture in females [57]. In dogs, increased internal femoral torsion increases risk of ACL rupture [58].

#### Neuromuscular factors

Develop of muscle fatigue includes hamstring reflex responses in women, but not men, and leads to increased anterior-posterior tibial translation [59]. In dogs, quadriceps atrophy may lead increased anterior tibial thrust [60].

#### Quadriceps angle and patellar tendon angle

In humans, the quadriceps angle (QA) is larger in females [61] and is weakly associated with increased risk of ACL rupture. In dogs, QA does not influence risk of ACL rupture [58]. Patellar tendon tibial shaft angle in humans is also a moderate predictor of ACL injury [62], and similar effects on ACL rupture risk in dogs may exist [63].

#### Genetic risk

Genetic risk influences the pathological mechanisms that lead to ACL rupture, but specific linkages are not defined. Humans with ACL rupture are twice as likely as control subjects to have a family member with ACL rupture [5]. Family members of bilateral ACL rupture patients have increased risk [64].

Candidate gene studies have implicated genes that influence ligament matrix properties [65]. Genetic effects that increase ligament laxity, reduce strength, promote fibroblast apoptosis, promote abnormal matrix remodeling, or influence synovial sheath inflammatory responses may all influence injury risk. Genome-wide association studies (GWAS) have identified ACL rupture candidate variants [66, 67]. Human ACL rupture heritability has not been estimated.

In high-risk dog breeds, ACL rupture is moderately heritable with a polygenic architecture [34]. Interestingly, Type I (*COL1A1*) and Type V collagen (*COL5A1*), and the large proteoglycan, aggrecan (*ACAN*), have been identified as risk genes in both species [34, 68–71].

#### Research opportunities

The epidemiology of non-modifiable risk factors has several shared features in humans and dogs such as internal femoral torsion/rotation and several areas of difference between the two species. Comparative research opportunities appear more limited in this area except for genetic research, where several ACL rupture risk genes that are shared between the two species have been identified to date. As larger epidemiological data sets become available, this may clarify the role of specific risk factors in the two species, such as relative tibial tuberosity width.

### Modifiable factors

#### Environmental factors

Environmental factors have not commonly been shown to be different between sexes [72]. In dogs, there are no specific data that suggest increased ACL rupture risk from a particular terrain [73].

#### Activity and neuromuscular training

Recent efforts to develop neuromuscular training programs for human athletes have shown promise in lowering ACL rupture rates [74]. Habitual activity is not a risk factor for ACL rupture in Labrador Retrievers, a high-risk breed [73].

#### Body mass index

Greater body mass index (BMI) is a risk factor for human ACL injury in some studies, especially when combined with increased PTS [75]. Obesity in dogs increases ACL rupture risk (OR ~ 2.1–3.8) [76, 77].

#### Research opportunities

Obesity is common in both humans and dogs and interactions between BMI and ACL rupture risk could represent another area of research that needs more investigation.

### Clinical presentation

#### Patient history and physical examination

Humans with ACL rupture typically present with knee pain, swelling, instability, and hemarthrosis. Increased anterior tibial translation and internal tibial rotation is often present [78]. The Lachman, anterior drawer, and pivot-shift tests are used to assess instability [79]. Dogs with ACL rupture typically present with lameness after a non-contact incident associated with normal activity. In dogs, limb muscle atrophy, knee effusion, medial periarticular fibrosis, and palpable anterior-posterior and internal rotational laxity are common findings [80]. Approximations of the Lachman, anterior drawer, and pivot-shift tests that are used in humans are also used to evaluate knee instability in dogs [80]. Knee fibrosis may reduce palpable joint laxity. In both species, anterior translation of the tibia with an ill-defined endpoint is indicative of ACL rupture. Anterior tibial translation while walking in ACL deficient dogs (~ 9.7-10 mm) is much greater than in ACL deficient humans (~ 3 mm) [78, 81].

#### Diagnostic imaging

**Fig. 2:**
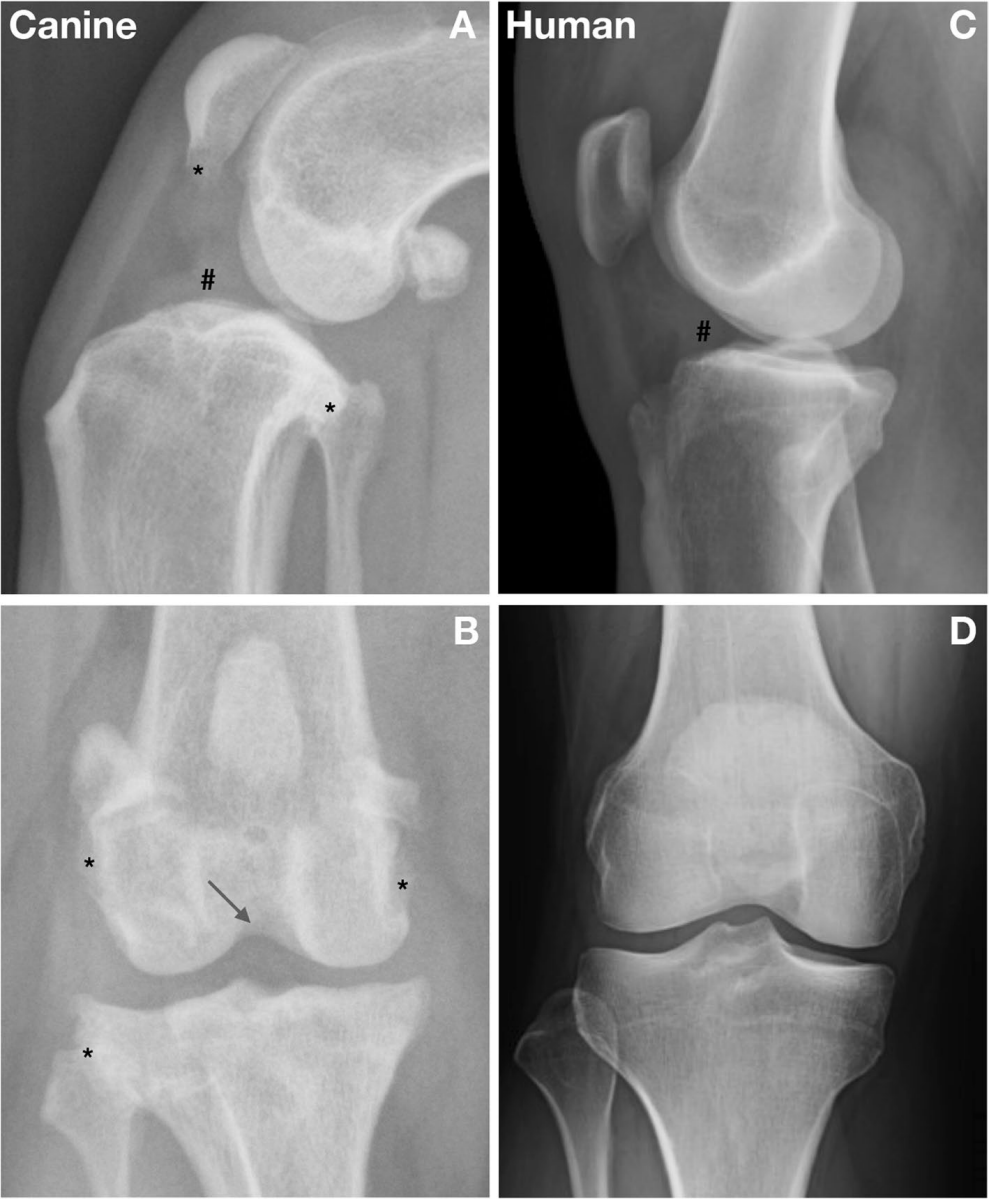
Radiographic features of anterior cruciate ligament (ACL) rupture in the dog and human. **A**,**B** Lateral and anterior-posterior (AP) views of the right knee of a dog with anterior crucitate ligament (ACL) rupture and palpable laxity. The presence of knee joint effusion (#), osteophytes (*) and some degree of intercondylar notch narrowing (arrow) are typical at diagnosis. **C**,**D** Lateral and AP radiographs of the right knee of a human demonstrating joint effusion (#). Secondary OA typically develops after ACL rupture in humans

Knee radiography is more sensitive than palpation for detection of effusion and OA. Avulsion fractures at ligament attachments may occur in both species. In dogs, subtle radiographic change may be present with obvious synovitis (Fig. 2). Radiographic knee effusion and OA predict progression of ACL rupture from incomplete to complete in dogs [33].

**Fig. 3:**
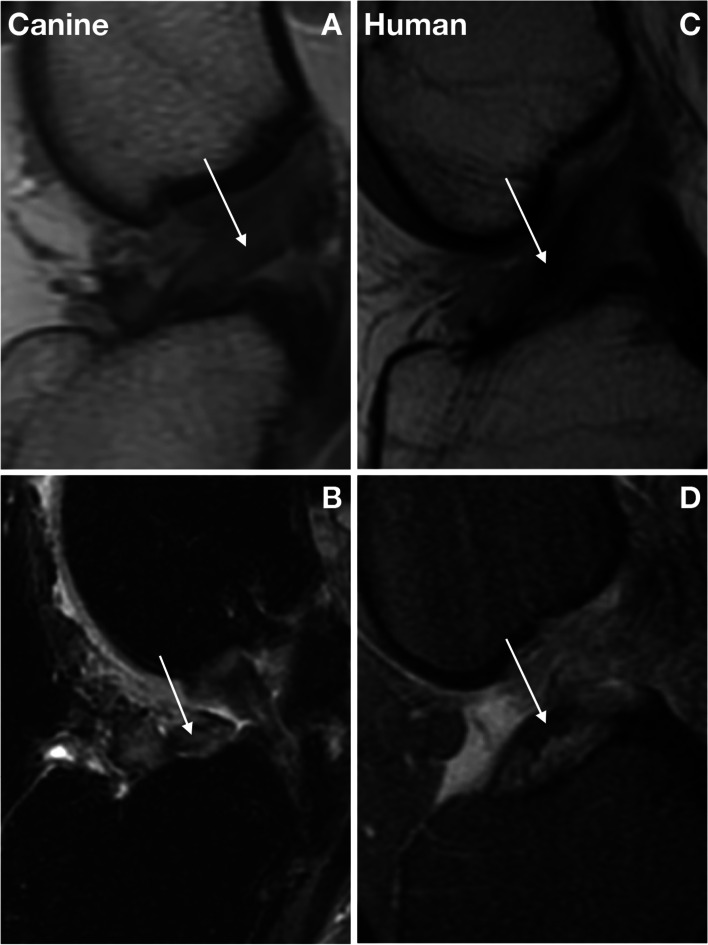
Magnetic resonance (MR) imaging of incomplete and complete anterior cruciate ligament (ACL) rupture in the dog and human. Both humans and dogs can present with incomplete or complete anterior cruciate ligament (ACL) rupture. **A** Sagittal proton density fast spin echo (FSE) magnetic resonance (MR) image of a stable canine knee with incomplete ACL rupture (arrow). **B** Sagittal T2 FSE CUBE image of a complete canine ACL rupture (arrow). In humans, incomplete ACL rupture develops gradually with subfailure fiber rupture similar to the dog. **C** Sagittal MR imaging illustrating incomplete human ACL rupture (arrow). **D** T2-weighted MR sagittal sequence demonstrating mid-substance complete ACL rupture (arrow). Images A and B in Fig. 3 are reproduced with permission from Wiley [82]

Magnetic resonance (MR) imaging is the best method for identifying ACL fiber tearing in both species, with isotropic 3 T sequences being advantageous [82]. Both humans and dogs with incomplete ACL ruptures have MR signal changes associated with fiber tearing (Fig. 3) that may not be detectable by physical examination, as well as signal changes in the synovium, meniscus, and subchondral bone [31, 82, 83].

Bone bruises identified by MR imaging are associated with ACL rupture in both species [84, 85]. Humans with ACL rupture often have bruising of femoral and tibial subchondral bone suggesting that landing on an extended knee with a flexion angle near full extension with increased valgus, internal tibial rotation, and anterior tibial translation is a risk factor for ACL rupture [84]. In dogs, bone bruises often occur by the intercondylar notch and the tibial eminences, likely reflecting high ACL strains [85].

#### Intraarticular findings from the knee joint

**Fig. 4:**
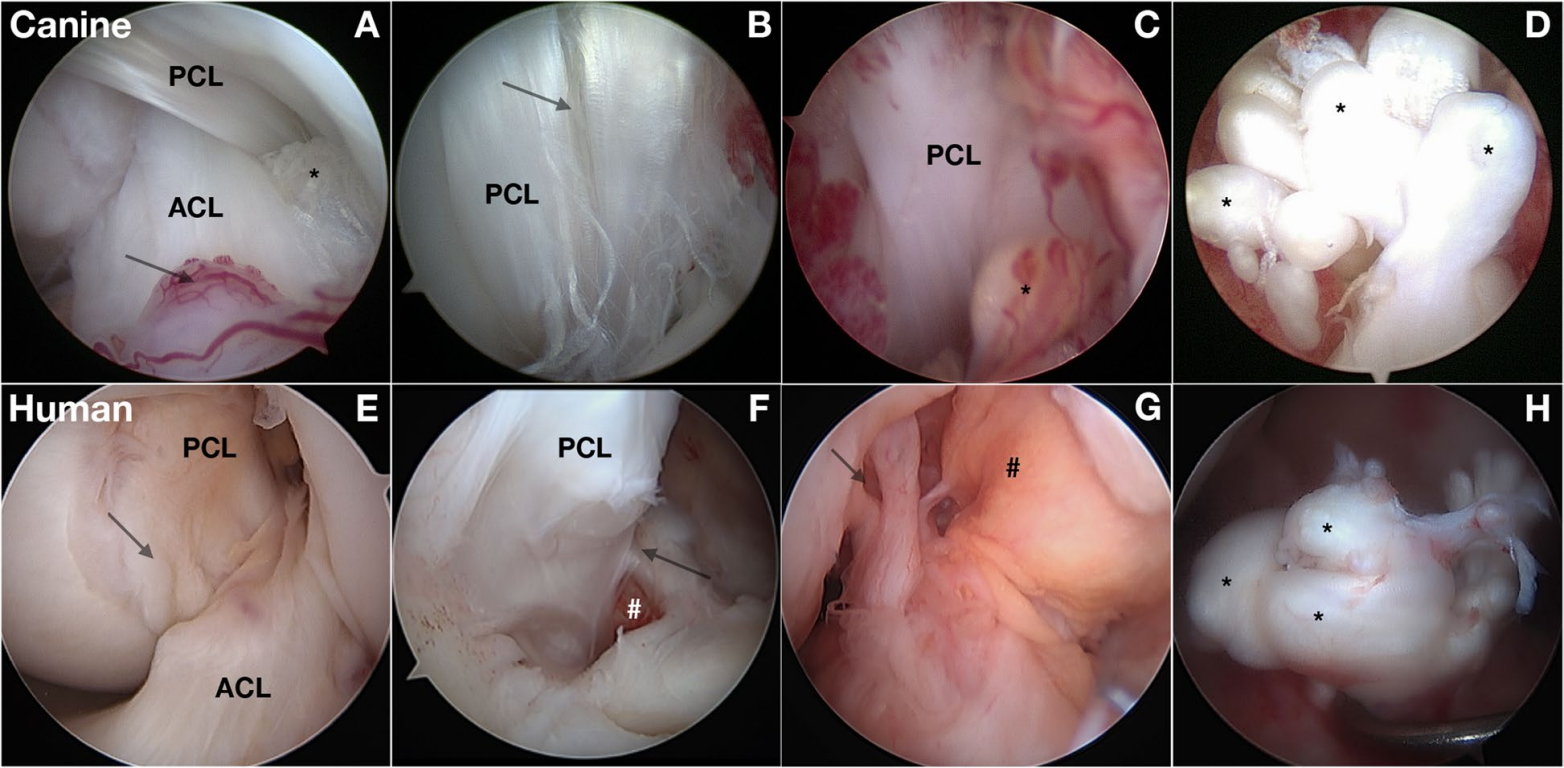
Intraarticular findings associated with anterior cruciate ligament (ACL) rupture in the dog and human. **A**-**D** Arthroscopic views of the intercondylar notch (ICN) in dogs with anterior cruciate ligament rupture (ACL) (*). **A **Fiber rupture often involves specific fiber bundles in the anteromedial bundle of the ACL (*). Associated synovitis is present (arrow). **B** Fiber rupture and splitting (arrow) of the posterior cruciate ligament (PCL) is also common. **C** With progressive fiber rupture, associated synovitis reflects hypertrophy, vascularity and inflammatory changes. The healing response in fiber bundles (*) is not successful. **D** View of the tibial attachment of a complete ACL rupture. A marked healing response in ruptured fiber bundles (*) leads to enlargement of ruptured fiber bundles. **E**-**H** Arthroscopic views of a human knee with ACL rupture. **E** The femoral ICN containing both ACL and PCL as they twist around each other with overlying synovium (arrow) is similar to dogs. Both species develop an associated synovial inflammatory response. **F** PCL fiber rupture (arrow) with adjacent synovitis and hemorrhage (#). **G** The ACL rupture can be seen with few fibers remaining attached to the femur (arrow) with synovitis (#) overlying the PCL. **H** The blunted end of ruptured ACL fibers at the tibial attachment, similar to the dog

In both species with suspected incomplete ACL rupture, arthroscopy typically confirms fiber rupture (Fig. 4) [31, 86]. In additional to ipsilateral knee synovitis, contralateral knee synovitis is often present in dogs [86]. PCL fiber rupture is often identified in dogs with incomplete or complete ACL rupture [86]. Softening and fibrillation of hyaline articular cartilage with osteophyte formation and meniscal damage is common in both humans and dogs with complete ACL rupture [86, 87]. Chondroid metaplasia and matrix degeneration of the ACL is similar in both species [88, 89].

#### Research opportunities

Studies of the progression of fiber rupture in patients with incomplete ACL rupture is limited in humans. The frequency and degree of PCL fiber rupture in human patients with ACL rupture is currently a gap in knowledge. The spontaneous canine ACL rupture model could provide new opportunities to advance fundamental knowledge of the pathogenesis of incomplete ACL rupture, particularly through serial imaging studies. Serial MR imaging could shed new light on development of bone bruises in ACL rupture patients. Such research would be further enhanced by associated molecular analyses of synovial fluid and cruciate ligament tissue.

### Treatment

The mainstay of conservative treatment is a regimented physical therapy program and medical management of OA. Canine ACL rupture and knee OA creates the opportunity for large animal model testing of intraarticular biological products [90].

Human and veterinary orthopaedic surgeons have used numerous surgical treatments for ACL rupture. In humans, primary repair and reconstruction have been the main intraarticular techniques. Recent research of bridge-enhanced primary repair for incomplete and complete ACL ruptures in humans has become more prevalent due to interest in lowering incidence of posttraumatic OA and preservation of native tissue proprioceptive and biomechanical function [2, 3]. Primary ACL repair in dogs often results in ACL laxity, fiber loss and OA [91]. ACL repair with an intraarticular graft has been used in both species but is rarely used in dogs currently because of poor outcomes, which is a limitation of the spontaneous canine ACL rupture model. Challenges with graft mechanical strength and fixation, cellular ingrowth, knee loading after surgery, disruption of ligament blood supply, and exposure to synovitis likely explain this difference.

A high tibial osteotomy or tibial plateau leveling osteotomy (TPLO) to reduce PTS offers the best outcome in dogs [18]. Humans with failed ACL reconstruction and PTSs > 13° may also benefit from tibial osteotomy along with ACL repair [92].

#### Research opportunities

Large populations of client-owned dogs undergo surgical treatment of ACL rupture each year. This animal population is an untapped resource regarding treatment related research, particularly progression of OA and studies of disease-modifying therapy.

### Impact on human health

#### Comparative diagnostic metrics

Most dogs are managed based on physical examination and radiographs. Knee arthroscopy is also commonly performed in humans and dogs. Longitudinal radiography and arthroscopy in dogs are easily accessible for research.

#### Availability of knee tissues

Knee synovial fluid arthrocentesis in dogs is easy to perform. This creates an opportunity for comparative studies of synovial fluid biomarkers. Identification of a biomarker that is unique to ACL rupture, rather than post-traumatic OA and investigation of molecular pathways of ACL degeneration could be improved through use of canine joint tissues. The shortened lifespan of dogs enables end-of-life studies.

#### Isolation of specific outcomes associated with surgical technique

Dogs with incomplete ACL ruptures may undergo TPLO for PTS reduction to prevent further ACL fiber rupture [15]. This provides opportunities for studying ligament fiber response and ACL healing. Outcomes of TPLO in dogs could help isolate the long-term impact of PTS reductions on OA progression [15]. Some human patients experience rotational instability after ACL reconstruction [93], which can also be seen in dogs. ALL reconstruction in humans [93] is analogous to extracapsular knee stabilization in dogs, which could be used as an animal model for ALL reconstruction. The role of knee synovitis in graft healing and associated knee laxity in humans is poorly understood. Studies of graft failure in the spontaneous canine ACL rupture model could yield new insight into mechanisms explaining poor graft healing.

#### Causal genetic variant discovery and genomic prediction of ACL rupture risk

Use of dogs for genetic investigation of human complex disease is advantageous [34]. Longer haplotype blocks allow GWAS to be performed with fewer markers and smaller sample sizes [34]. Development of polygenic risk scoring for ACL rupture risk prediction is enabled by dog studies [94].

## Conclusions

Many epidemiological features of ACL rupture are comparable between humans and dogs. Given the high incidence in many different breeds of dog, there is a very large study population available for comparative research. The greatest limitations of the spontaneous canine ACL rupture model are different knee biomechanics related to quadrupedal locomotion and differences in surgical treatment. OA progression is faster in dogs, enabling disease course observation over a shorter time. Sharing of ACL rupture genetic variants with humans has not been specifically investigated to date. Use of client-owned dogs as a preclinical animal model for studies of ACL rupture biology and response to treatment promises advances in translational One Health research.

## Data Availability

Not applicable.

## Abbreviations

ACL: Anterior cruciate ligament
OA: Osteoarthritis
PCL: Posterior cruciate ligament
ICN: Intercondylar notch
PTS: Posterior tibial slope
ALL: Anterolateral ligament
QA: Quadriceps angle
GWAS: Genome-wide association study
BMI: Body mass index
TPLO: Tibial plateau leveling osteotomy
IFP: Infrapatellar fat pad
MFC: Medial femoral condyle
LDE: Long digital extensor
AP: Anterior-posterior
MR: Magnetic resonance
FSE: Fast spin echo
